# Shift the paradigm to shift the weight: obesity care in the community

**DOI:** 10.3399/bjgp24X738465

**Published:** 2024-05-31

**Authors:** Hannah O’Hara, Alexander Dimitri Miras

**Affiliations:** Clinical Lecturer, Centre for Public Health, School of Medicine, Dentistry and Biomedical Sciences, Queen’s University Belfast, Belfast; Castlereagh Medical Centre, Belfast.; Professor of Endocrinology, Ulster University, School of Medicine, Faculty of Life and Health Sciences, Derry.

## Introduction

Obesity is a chronic relapsing condition characterised by abnormal or excessive accumulation of adipose tissue that presents a risk to health. It is associated with an increased risk of other chronic conditions including type 2 diabetes (T2DM), cardiovascular disease, renal and liver disease, musculoskeletal problems, and cancer.^1^ It can impact a person’s quality of life, wellbeing, and lifespan. Obesity is a complex condition, with a multitude of biological, social, environmental, and commercial factors, as well as food production and consumption processes, contributing to its development.^2^ One in four adults in the UK is living with obesity with comparable figures seen in children, and recent decades have seen an increase in prevalence.^3^^,^^4^

## Current weight management services

The National institute for Health and Care Excellence (NICE) updated its guidelines on the management of obesity in 2023.^5^ Weight management services were previously offered in tiers according to severity and complexity of disease, with Tiers 1 and 2 addressing population-level public health measures and short-term primary care-based services focusing on dietary and lifestyle factors, respectively. Tier 3 services offered pharmacotherapy in addition to intensive behavioural interventions for those with complex, refractory, or severe disease, while bariatric surgery was delivered within Tier 4 services. Engagement with one tier was necessary before onward referral to the next.

Evidence does not support using bariatric surgery as a last resort and changes to NICE guidance removed the requirement for exhaustion of non-surgical measures prior to consideration of surgery.^5^ The update also considered Tiers 3 and 4 together as specialist obesity services composed of a multidisciplinary team (MDT) including physicians, specialist nurses, specialist dieticians, psychologists, psychiatrists, and physiotherapists. Access to these services is hugely variable across the UK, with only 21% of clinical commissioning groups in England offering these services in 2014–2015 and one devolved nation (Northern Ireland) having no access whatsoever.^6^ It is hoped that the recent amendments will reduce health inequalities resulting from unequal access to specialist obesity services but, for those living in areas with no access to bariatric surgery, there is potential for inequities to widen. Undoubtedly, the variability in access to bariatric surgery needs to be addressed. However, in focusing on access to surgery, it is imperative that we do not neglect expansion of non-surgical obesity services, paying particular attention to areas with little or no resource.

## Could a community-based approach offer a solution?

Specialist obesity services are currently almost exclusively delivered in secondary care, but managing a condition affecting more than 20% of the population in this setting is impractical. Conditions with comparable prevalence, such as hypertension, are managed in primary care with only the most complex patients referred to secondary care. The development and implementation of Integrated Care Systems offer an important opportunity to transform how obesity services are delivered. If the concept of integrated care is embraced and actioned as intended, obesity care could be an authentically patient-centred service, with dissolution of silos allowing coordinated effective care for those who need it. This notion has already been demonstrated in diabetes care, with MDTs delivering community-based specialist diabetes care addressing the holistic needs of the patient. There is significant overlap between the components of diabetes and obesity care, and adaptation of existing pathways would be a viable option. The NICE definition of a specialist obesity service recognises its potential to be a specialist primary, community, or secondary care-based MDT. Primary care is embedded within the community, maintaining an in-depth knowledge of the population it serves, and is uniquely placed to deliver obesity care at scale.^7^

NICE recommends several management options for obesity within a specialist service, as described below. We propose that a substantial proportion of this service could be delivered in a primary care or community-based setting, in combination with tailored psychological support.

### Behavioural modification

People living with obesity should be supported to increase their activity levels and improve eating behaviours and diet quality, with significant health benefits to be gained regardless of whether weight loss occurs. Increased activity can be achieved through active travel, for example, or through supervised exercise programmes. Potential dietary approaches include small energy deficits, intermittent fasting, high protein and low carbohydrate consumption, meal replacement, and adoption of a Mediterranean diet. Evaluation of response is crucial, and trial of several approaches may be necessary to find the most effective for the individual. Behavioural modifications to diet and physical activity behaviours could be effectively delivered by GPs using brief interventions or motivational interviewing, for example, or by community-based MDTs comprising dieticians, physical activity specialists, and psychologists. The availability of digitally delivered services further expands the potential for community-based care.^8^

It is recognised that multicomponent interventions incorporating behaviour change strategies that target physical activity and eating behaviours, as well as dietary quality, are likely to be most successful.^9^ In formulating these multicomponent interventions, it is important to be cognisant of the patient’s preferences and social and cultural circumstances as these will influence activity and dietary options. The primary care team is optimally placed to navigate these subtleties alongside the patient, within their own community. An appreciation of the local context and a good working knowledge of local support systems from voluntary support organisations would allow optimisation of individualised behavioural modification interventions.

### Pharmacotherapy

A number of pharmacotherapy options are available for the management of obesity (Table 1). The glucagon-like peptide 1 (GLP-1) receptor agonist semaglutide 2.4 mg (Wegovy) has been shown to induce a mean body weight loss of 16%, significantly more than the previously available GLP-1 analogue liraglutide 3.0 mg (Saxenda) and the lipase inhibitor Orlistat.^10^ The NICE guidance on the use of Saxenda limited its use to secondary care but the recent guidelines on the use of Wegovy enable increased flexibility, allowing it to be offered within specialist obesity service without dictating the clinical setting. In 2023, the UK government announced a £40 million pilot focusing on how semaglutide can be used safely and effectively in a community setting. Execution of this body of work will be instrumental in guiding implementation and future service provision.

**Table 1.** Pharmacological options for the treatment of obesity, adapted from the National Institute for Health and Care Excellence guidance on the identification, assessment, and management of obesity^5^

**Pharmacological agent**	**Setting**	**Patient eligibility**
Liraglutide 3.0 mg daily (GLP-1 receptor agonist Saxenda)	To be prescribed in secondary care by a specialist weight management service	BMI of: 35 kg/m^2^ or more or 32.5 kg/m^2^ for members of minority ethnic groups known to be at equivalent risk of the consequences of obesity at a lower BMI than the Caucasian population AND non-diabetic hyperglycaemia (HbA1c level of 42–47 mmol/mol or a fasting plasma glucose of 5.5–6.9 mmol/l AND high risk of CVD based on risk factors such as hypertension and dyslipidaemia
Semaglutide 2.4 mg weekly (GLP-1 receptor agonist Wegovy)	To be prescribed within a specialist weight management service	BMI of: 35 kg/m^2^ or more or 30 kg/m^2^ to 34.9 kg/m^2^ and meet the criteria for referral to specialist weight management service Use lower BMI thresholds (reduce by 2.5 kg/m2 for people from South Asian, Chinese, other Asian, Middle Eastern, Black African, or African Caribbean family backgrounds) AND At least 1 weight-related comorbidity
Orlistat 120 mg with main meals (lipase inhibitor)	Primary care	BMI of: 30 kg/m^2^ or more or 28 kg/m^2^ or more with associated risk factors

*BMI = body mass index. CVD = cardiovascular disease. GLP = glucagon-like peptide 1.*

**Figure 1.**
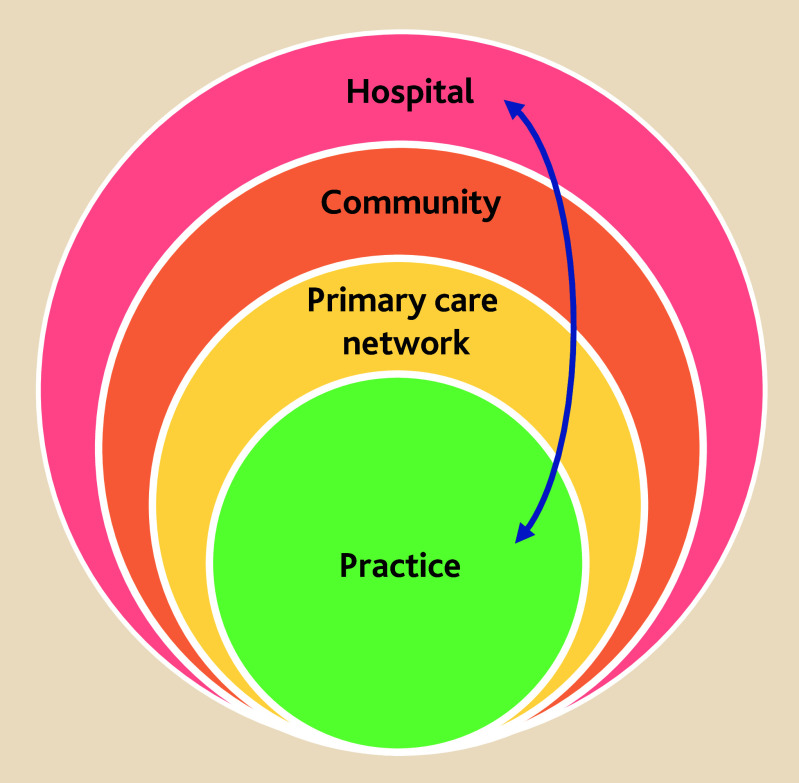
The principles of a good model for obesity care, adapted from recommendations from Diabetes UK and the Primary Care Diabetes Society. Source: https://www.diabetes.org.uk/for-professionals/improving-care/good-practice/primary-and-community-care/pcn-delivery-of-care.^11^ © Diabetes UK. Used with permission.

GLP-1 receptor agonists have been used to treat diabetes for more than 16 years and have a well-established safety profile. They are confidently prescribed within a primary care setting in the management of T2DM, and their use could rationally be expanded to obesity care in this setting or within primary care networks (PCNs). The required monitoring is not intensive and, in the event of serious adverse events, discontinuation is straightforward. With appropriate training and support from specialist nurses and pharmacists with an interest in obesity, GPs could deliver this service in a manner analogous to delivery of diabetes services.^11^ Figure 1 shows our proposed model of obesity care, based on the model of diabetes care from Diabetes UK and the Primary Care Diabetes Society recommendations, with a great majority of patients expected to be able to be managed in a community setting without the need for secondary care involvement.

### Bariatric surgery

While bariatric surgery can only be delivered in a hospital setting, much of the pre- and post-operative care does not require the same facilities. Bariatric surgery can be offered to those with a BMI of 40 kg/m^2^ or more, or at lower thresholds where there is a significant health condition such as T2DM or cardiovascular disease that could be improved with weight loss, or in those with a higher-risk ethnic background. Pre-operative assessment, management of comorbidities, and assessment of nutritional status could be achieved within the context of a community-based MDT following initial assessment by the patient’s GP. Post-operative lifelong support could, similarly, be provided locally by the patient’s GP, with support from MDT members, to ensure optimal nutritional status, effective medication review, and psychological support under shared-care agreements with secondary care. As Figure 1 demonstrates, the fundamental characteristic of such an obesity care model is the seamless movement of patients from primary to secondary care *and back*.

## Barriers to delivering community-based obesity services

Identification and assessment of patients with obesity is regularly carried out within the primary care setting, but several factors limit GPs’ ability to further address the issues under consideration. Fear of damaging the doctor–patient relationship, as well as self-perceived limited understanding of obesity management, have been found to hinder constructive dialogue.^12^ This lack of confidence is not surprising: despite the relationship between nutrition and health, nutrition education is lacking in undergraduate medical education, with as few as 26% of junior doctors feeling confident in discussing the topic with patients.^13^ Geographical variability of specialist services also impedes discussion as locally available treatment options may be limited.

A common misconception among the public and many clinicians is that the problem of obesity could be solved if only people ate less and moved more. The pervasiveness of weight bias has fuelled this narrative and evidence suggests that the weight stigma contributes to increased morbidity and mortality, independent of weight or BMI.^14^^,^^15^ While it is true that, at a fundamental level, obesity results from a shift in a person’s homeostatic regulation of energy balance, a multitude of variables contribute to the development of obesity at an individual level. Acknowledging the role of weight bias and addressing it is necessary to begin to develop services that will meaningfully impact the health and lives of people living with obesity. Increased training at undergraduate and postgraduate level is also required to equip future GPs with the knowledge and skills to deliver obesity care.

Despite obesity being a chronic disease, it is not recognised as such within the frameworks that aim to remunerate practices for providing high-quality chronic disease management. Within the Quality and Outcomes Framework, obesity falls under the ‘public health’ rather than ‘clinical’ domain. While required to maintain an obesity register, there is no requirement to instigate subsequent management and ongoing review of these patients, even though effectively managing obesity would improve outcomes in many of the conditions listed within the ‘clinical’ domain, including T2DM. There must be a redistribution of both focus and funding towards obesity management within the primary care setting, rather than merely prevention and identification.

In developing primary care-based obesity care, attention must be paid to the needs of the workforce. Lack of time and resource are cited as barriers to engaging in weight management conversations with patients^12^ and, while few practitioners would deny the need for expansion of weight management services, many would be reluctant to take on additional work at a time when resources are already overstretched, even with sufficient remuneration. Practice closures are at an all-time high, with high workload and recruitment problems commonly cited as causative factors. The NHS Long Term Plan promised increased focus on recruitment of staff, and this, alongside dedicated funding and resources, will be crucial in ensuring delivery of high-quality community-based obesity care.^7^

## Conclusion

With appropriate training, a significant proportion of obesity care could be delivered by GPs at a practice level. Additional components of the obesity care model can be delivered by MDTs, which are being increasingly incorporated into general practices and which are well established within PCNs. Further development and expansion of these teams would allow effective and efficient delivery of the majority of components of a specialist obesity management service within a primary care or community setting. When considering strategies for treating obesity in any individual, it is crucial to maintain a person-centred approach that focuses on health promotion, and which facilitates ease of transition between these various components. The development of a coordinated approach in collaboration with secondary care providers would expand access to effective non-surgical obesity management services for those who require it while removing barriers to bariatric surgery where appropriate. There are several factors that would mediate successful expansion of a primary care-delivered service, including appropriate remuneration, resources, recruitment, and training. However, if specialist obesity services could be embedded within primary care, it could vastly expand access to much needed services across the UK, simultaneously reducing existing health inequalities.
